# Complete Genome Sequence of *Paracoccus yeei* TT13, Isolated from Human Skin

**DOI:** 10.1128/genomeA.01514-17

**Published:** 2018-01-18

**Authors:** Jae Yun Lim, Ingyu Hwang, Munkhtsatsral Ganzorig, Shivalika Pokhriyal, Rajni Singh, Kyoung Lee

**Affiliations:** a Department of Agricultural Biotechnology, Seoul National University, Seoul, South Korea; b Department of Bio Health Science, Changwon National University, Changwon, South Korea; c Amity Institute of Microbial Biotechnology, Amity University, Noida, India

## Abstract

*Paracoccus yeei* TT13 was isolated from human skin because of its ability to degrade propylene glycol. Here, we present the whole-genome sequence of this strain; it possesses one 3.58-Mb chromosome and six plasmids. TT13 genome analysis indicated that this bacterium has denitrification potential.

## GENOME ANNOUNCEMENT

*Paracoccus yeei* is a Gram-negative coccobacillus which has been isolated from diverse natural environments, including human skin (1). This strain is also an opportunistic human pathogen that has occasionally been linked with peritonitis and bacteremia (2, 3). One complete genome sequence of *P. yeei*, that of FDAARGOS_252, which was isolated from a female with abdominal pain, is presented in the NCBI database. Here, we report the complete genome sequence of *P. yeei* strain TT13, isolated from human skin. This strain can grow using propylene glycol, a common ingredient in cosmetics, as its sole source of carbon and energy.

*P. yeei* TT13 (KCTC 52861) was isolated from the forehead of a male college student by swab sampling and direct streaking on minimal salts basal (MSB) medium containing 0.01% yeast extract and 0.1% propylene glycol (4). This strain requires growth factors for growth in minimal basal medium. Ethical approval for subject sampling was granted by the Changwon National University ethics committee. Total DNA of the cultured cells was extracted using the phenol extraction method (5). Whole-genome sequencing was performed on the RS II platform (PacBio) using 20-kb SMRTbell template libraries (National Instrumentation Center for Environmental Management [NICEM], Seoul National University). The obtained reads, with 178-fold genome coverage, were assembled *de novo* using Hierarchical Genome Assembly Process (HGAP) 3.0. Complete genome sequences were obtained by bioinformatics analysis, as previously described (6). Gene predictions and annotations were provided by NCBI using the Prokaryotic Genome Annotation Pipeline (7). The SEED subsystem via the Rapid Annotation using Subsystems Technology (RAST) server was used for functional categorization of the predicted proteins (8).

The *P. yeei* TT13 genome comprises a 3,588,087-bp chromosome (G+C content, 67.3%) and six plasmids (pTT13-1, pTT13-2, pTT13-3, pTT13-4, pTT13-5, and pTT13-6, comprising 327,671, 225,271, 167,927, 172,023, 114,679, and 24,567 bp, respectively). The chromosome contains 3,489 predicted coding genes, 51 tRNA genes, and 3 rRNA operons. Plasmids pTT13-1, pTT13-2, pTT13-3, pTT13-4, pTT13-5, and pTT13-6 carry 299, 216, 168, 137, 110, and 24 coding genes, respectively. The presence of genes encoding four lactate dehydrogenases and the absence of the *pdu* operon indicated that propylene glycol is catabolized to pyruvate but not to propionate (9). In addition, the genes encoding the proteins that catabolize 3,4-dihydroxybenzoate through the β-keto-adipate pathway were found dispersed in the chromosome and pTT13-2 plasmid. Interestingly, like the genome of *Paracoccus denitrificans* PD1222, the TT13 genome was shown to contain clusters of denitrification genes (*nar*, *nor*, and *nos*) and genes related to the Calvin-Benson cycle and the RuBisCO genes (*rbcLS*) for autotrophic growth (10). By judging the suite of such genes for environmental succession, it is supposed that *P. yeei* TT13 originated from the surrounding environment. Investigation of the *P. yeei* TT13 genome will help ascertain the cellular and catabolic adaptations of this bacterium that have enabled it to colonize human skin.

### Accession number(s).

The complete genome sequence of the *P. yeei* TT13 strain was deposited at GenBank under the accession numbers CP024422 (chromosome) and CP024423 to CP024428 (pTT13-1, pTT13-2, pTT13-3, pTT13-4, pTT13-5, and pTT13-6, respectively).
